# Ганглионейроматоз кишечника как ранняя экстраэндокринная манифестация множественной эндокринной неоплазии 2B типа (МЭН 2B)

**DOI:** 10.14341/probl13302

**Published:** 2023-06-15

**Authors:** Ю. В. Аверьянова, Н. Ю. Калинченко, Д. Н. Бровин, Е. Е. Петряйкина, А. Н. Тюльпаков

**Affiliations:** Российская детская клиническая больница ФГАОУ ВО «РНИМУ им. Н.И. Пирогова»; Национальный медицинский исследовательский центр эндокринологии; Национальный медицинский исследовательский центр эндокринологии; Российская детская клиническая больница ФГАОУ ВО «РНИМУ им. Н.И. Пирогова»; Российская детская клиническая больница ФГАОУ ВО «РНИМУ им. Н.И. Пирогова»; Медико-генетический научный центр им. академика Н.П. Бочкова

**Keywords:** множественная эндокринная неоплазия тип 2B, медуллярная карцинома, ганглионейроматоз желудочно-кишечного тракта, RET

## Abstract

Множественная эндокринная неоплазия 2B типа (МЭН 2B) является редким вариантом наследственных опухолевых синдромов, обусловленным герминальными мутациями в гене протоонкогена RET. Одним из компонентов синдрома являются множественные невриномы, раннему выявлению которых не всегда уделяется должное внимание. Нами представлено описание случая МЭН 2B, манифестировавшего в первые месяцы жизни проявлениями ганглионейроматоза кишечника. Заболевание дебютировало хроническими запорами, в том числе с эпизодами кишечной непроходимости, потребовавшими повторных хирургических вмешательств. МЭН 2B был заподозрен в возрасте 15 лет. На момент диагностики отмечалось повышение уровня кальцитонина в сыворотке (1041 пг/мл, норма <9,5 пг/мл), а также определялся узел в щитовидной железе (1,3×1,0×1,2 см, TIRADS 5), в последующем верифицированный как новообразование из C-клеток. При ДНК-диагностике в гене RET выявлен типичный для МЭН 2B патогенный вариант p.Met918Thr. Данных за феохромоцитому на момент обследовании не получено. Пациенту проведена тиреоидэктомия с лимфаденэктомией. Обсуждаются трудности ранней диагностики спорадических случаев МЭН 2В в связи с неспецифичностью гастроинтестинальных проявлений заболевания.

## Abstract (en)

Multiple endocrine neoplasia type 2B (MEN 2B) is a rare variant of hereditary tumor syndromes caused by germinal mutations in the proto-oncogene RET. One of the components of the syndrome is multiple neurinomas, the early detection of  which is not always given due attention. We present a description of the case of MEN 2B, manifested in the first months of life by intestinal ganglioneuromatosis. The disease presented with chronic constipation, including episodes of intestinal obstruction that required repeated surgical interventions. MEN 2B was suspected at the age of 15. At the time of diagnosis, an increase in serum calcitonin levels was noted (1041 pg/ml, norm <9.5 pg/ml), and a node in the thyroid gland was also determined (1,3*1,0*1,2 see, TIRADS 5), subsequently verified as a neoplasm of C-cells. By DNA analysis, a pathogenic variant p.Met918Thr, typical for MEN2 B, was detected in the RET gene. No signs of pheochromocytoma were found at the time of investigation. The patient underwent a thyroidectomy with lymphadenectomy. The difficulties of early diagnosis of sporadic cases of MEN 2B due to the nonspecificity of gastrointestinal manifestations of the disease are discussed.

## ВВЕДЕНИЕ

Множественная эндокринная неоплазия 2B типа (МЭН 2B) является редким вариантом наследственных опухолевых синдромов, обусловленным герминальными мутациями в гене протоонкогена RET. Помимо фео­хромоцитомы и медуллярной карциномы щитовидной железы (МКЩЖ), являющихся проявлениями МЭН 2A и 2B, вариант 2B характеризуется также множественными невриномами и нередко марфаноидным фенотипом. Выявление эндокринных компонентов синдрома МЭН 2B, как правило, не вызывает трудностей. В специализированных центрах доступны современные методы лабораторной и инструментальной диагностики, позволяющие детектировать гормонально-активные образования на ранних этапах и осуществлять мониторинг пациентов в процессе лечения. Детекция герминальных мутаций в гене RET используется для выбора хирургической тактики при МКЩЖ, причем выявление типичных для МЭН 2B патогенных вариантов является показанием для операции в наиболее ранние сроки [1].

Что касается неэндокринных компонентов синдрома, то они могут быть не столь очевидны и сами по себе редко являются поводом для подозрения на МЭН 2B и проведения уточняющей диагностики. В настоящей статье нами представлено описание случая МЭН 2B, манифестировавшего в первые месяцы жизни проявлениями ганглионейроматоза кишечника.

## ОПИСАНИЕ КЛИНИЧЕСКОГО СЛУЧАЯ

Девочка, на текущий момент возраст 16 лет 3 мес.

От 2-й беременности, протекавшей без осложнений. Роды 1-е, срочные, вес при рождении 4050 г, длина 54 см. Наследственность: мать — рак грудной железы (операция).

С рождения у ребенка отмечены запоры (неотхождение мекония в 1-е сутки жизни) и увеличение размеров живота. В течение первого года жизни трижды госпитализирована в инфекционный стационар по поводу кишечной инфекции.

1 год 3 мес: кишечная непроходимость, экстренная операция — ревизия брюшной полости, наложена колостома на восходящий отдел толстой кишки. На операции выявлено расширение толстой кишки на всем протяжении, что расценено как болезнь Гиршпрунга.

1 год 7 мес: повторная лапаротомия, при гистологическом исследовании — нейронная дисплазия толстой кишки.

3 года 7 мес: релапаротомия, колэктомия, формирование илеоректального анастомоза.

5–10 лет: повторные бужирования суженного анастомоза без стойкого эффекта.

11 лет — имплантация туннелированного катетера в систему нижней полой вены с целью проведения парентерального питания, лечение осложнено тромбозом. Катетер удален через 6 мес.

12 лет 8 мес: релапаротомия, разобщение илеоректального анастомоза, выведение терминальной илеостомы; по данным гистологии установлен ганглионейроматоз толстой и тонкой кишки, с более выраженными изменениями в толстой кишке.

15 лет 3 мес: заподозрен МЭН 2B; УЗИ щитовидной железы — узловое образование размерами 1,3×1,0×1,2 см, TIRADS 5; кальцитонин в крови — 1041 пг/мл (норма <9,5 пг/мл); пункционная биопсия — новообразование щитовидной железы из С-клеток (диагностическая категория Bethesdа VI), метастатические изменения ткани лимфатического узла; кальцитонин в смыве иглы при биопсии лимфоузла >2000 пг/мл. ДНК-анализ (лимфоциты крови пробанда) — патогенная гетерозиготная мутация в гене RET (NM 020975.6) c.2753T>C (p.Met918Thr); у матери изменений в гене RET не выявлено.

15 лет 4 мес: рост — 134,2 см (SDS=-4,6), вес — 32,5 кг (SDS индекса массы тела -0,9), марфаноподобный фенотип отсутствует, «полные» губы, деформация стоп по типу pes cavus, АД — 100/70 мм рт. ст., половое развитие по Таннеру B2P2. УЗИ надпочечников: патологии не выявлено. Экскреция метанефринов с мочой: метанефрин — 411 мг/сут (25–312), норметанефрин — 323 мг/сут (35–445).

15 лет 5 мес: 2-этапная операция — тиреоидэктомия с центральной и боковой лимфаденэктомией справа, боковая лимфаденэктомия слева; назначен левотироксин 50 мкг/сут.

## ОБСУЖДЕНИЕ

Впервые термин «МЭН 2B» был упомянут в статье G. Chong и соавт. в 1975 г. [2]. Между тем сочетание основных компонентов синдрома (феохромоцитома, МКЩЖ и множественные невриномы) было описано еще в 1966 г. Е. Williams и D. Pollock [3], и несколькими годами позже был представлен случай со схожей симптоматикой и марфаноидным фенотипом [4].

Заболевание обусловлено герминальными мутациями в гене RET, что впервые было показано R. Hofstra и соавт. [5], выявившими миссенс-вариант Met918Thr у всех обследованных ими 9 пациентов с МЭН 2B. Мутация Met918Thr затрагивает C-концевой тирозинкиназный домен RET и обнаруживается при МЭН2B в подавляющем большинстве случаев, однако описаны и другие редкие варианты (Ser904Cys, Ser922Tyr), локализующиеся в том же функциональном домене белка [6][7].

Изменения желудочно-кишечного тракта как компонент синдрома МЭН 2B были описаны еще в ранних публикациях, посвященных данному заболеванию. J. Carney и соавт. проанализировали 16 случаев синдрома МЭН 2B, диагностированных в клинике Мейо, и документировали клинические и (или) морфологические изменения у всех 16 пациентов [8]. Авторы констатировали, что морфологические признаки ганглионейроматоза могут определяться на протяжении всего желудочно-кишечного тракта. Обращал на себя внимание также тот факт, что гастроинтестинальная симптоматика в 10 из 16 случаях отмечалась уже на первом году жизни. Типичными жалобами были хронические запоры (у 4 потребовавшие хирургического вмешательства), боли и вздутие живота. Показательно, что у 12 из 16 пациентов именно эти симптомы доминировали в клиническом течении и предшествовали выявлению эндокринных компонентов синдрома [8].

В структуре синдрома МЭН 2B характеризуется наиболее агрессивным течением, и для предотвращения метастазирования МКЩЖ рекомендовано проведение профилактической тиреоидэктомии в ранние сроки (в первые месяцы жизни) [1]. Если речь не идет уже об установленных случаях заболевания в семье, когда тестирование на мутацию Met918Thr может быть проведено уже при рождении, заподозрить диагноз в раннем возрасте сложно, и симптомы поражения желудочно-кишечного тракта (ганглионейромы слизистых полости рта и хронические запоры) могут быть единственным поводом для специализированного обследования. Несмотря на большой опыт, накопленный на протяжении более чем 50 лет с момента первого описания МЭН 2B, ранняя диагностика заболевания по-прежнему сопряжена с определенными трудностями. G. Mirra и соавт. представили описание 3 детей (17 мес, 17 мес и 6 лет), поводом для обследования которых послужил комплекс неспецифичных жалоб, при этом у всех 3 отмечались запоры [9]. У 2 младших детей (17 мес) МЭН 2B был диагностирован случайно при ­проведении NGS-­тестирования на синдромальную патологию. У третьего ребенка ген RET был проанализирован прицельно, но уже после выявления значимого повышения уровня кальцитонина в крови [9].

В представленном нами наблюдении в семейном анамнезе не было указаний на синдром МЭН, и, учитывая данные анализа гена RET у матери, можно с большой вероятностью говорить о возникновении у пробанда мутации Met918Thr de novo. Последнее не является редкостью и, по данным литературы, отмечается приблизительно в 50% случаев МЭН 2B [10]. Отсутствие семейной настороженности, безусловно, явилось одной из причин поздней диагностики МЭН 2B у обследованного нами пациента, хотя проявления гастроинтестинального компонента синдрома, вероятно, присутствовали уже при рождении (мекониальный илеус). Последующее течение процесса и признаки болезни Гиршпрунга по результатам биопсии кишки также могли быть основанием для исключения патологии, связанной с геном RET. К сожалению, МЭН 2B был диагностирован уже при наличии определяемого по УЗИ узлового образования в щитовидной железе, высокого уровня кальцитонина в крови и в смывах из биоптатов лимфатических узлов. Последующее наблюдение позволит оценить успешность радикальной тиреоидэктомии. Параллельно будет продолжено наблюдение за состоянием надпочечников. На момент написания статьи отмечено умеренное повышение уровня метанефрина при отсутствии визуализирующихся образований надпочечников.

## ЗАКЛЮЧЕНИЕ

Таким образом, представленный нами случай и литературные данные свидетельствуют о ранней манифестации ганглионейроматоза желудочно-кишечного тракта при синдроме МЭН 2B. Учитывая, что, с одной стороны, гастроинтестинальная симптоматика при данном заболевании неспецифична, а с другой — агрессивность течения МКЩЖ диктует необходимость установления диагноза в максимально ранние сроки, считаем целесообразным проведение скрининга на частую мутацию в гене RET (Met918Thr) у детей с хроническими запорами неясной этиологии.

## ДОПОЛНИТЕЛЬНАЯ ИНФОРМАЦИЯ

Источники финансирования. Работа выполнена по инициативе авторов без привлечения финансирования.

Конфликт интересов. Авторы декларируют отсутствие явных и потенциальных конфликтов интересов, связанных с содержанием настоящей статьи.

Участие авторов. Все авторы одобрили финальную версию статьи перед публикацией, выразили согласие нести ответственность за все аспекты работы, подразумевающую надлежащее изучение и решение вопросов, связанных с точностью или добросовестностью любой части работы.

Согласие пациента. Пациент добровольно подписал информированное согласие на публикацию персональной медицинской информации в обезличенной форме.
